# Supplementary material for: Architecture of the yeast Elongator complex
Source: EMBO Rep. 2016 Dec 14;18(2):264–79. doi: 10.15252/embr.201643353 (PMC5286394; doi:10.15252/embr.201643353)
Supplement: Supplementary file 1 — Appendix [file EMBR-18-264-s001.pdf]

Appendix Figure and Tables

Dauden et al.

Architecture of the yeast Elongator complex

Appendix Figure S1
Sample preparation and crosslinking validation

Appendix Figure S2
Validating the Elp1-Elp3-Elp4 interaction network

Appendix Figure S3
Conservation and charge distribution of holoElongator

Appendix Table S1
Crystallographic data collection, phasing and refinement statistics

Appendix Table S2
List of highly confident crosslinks identified in this work

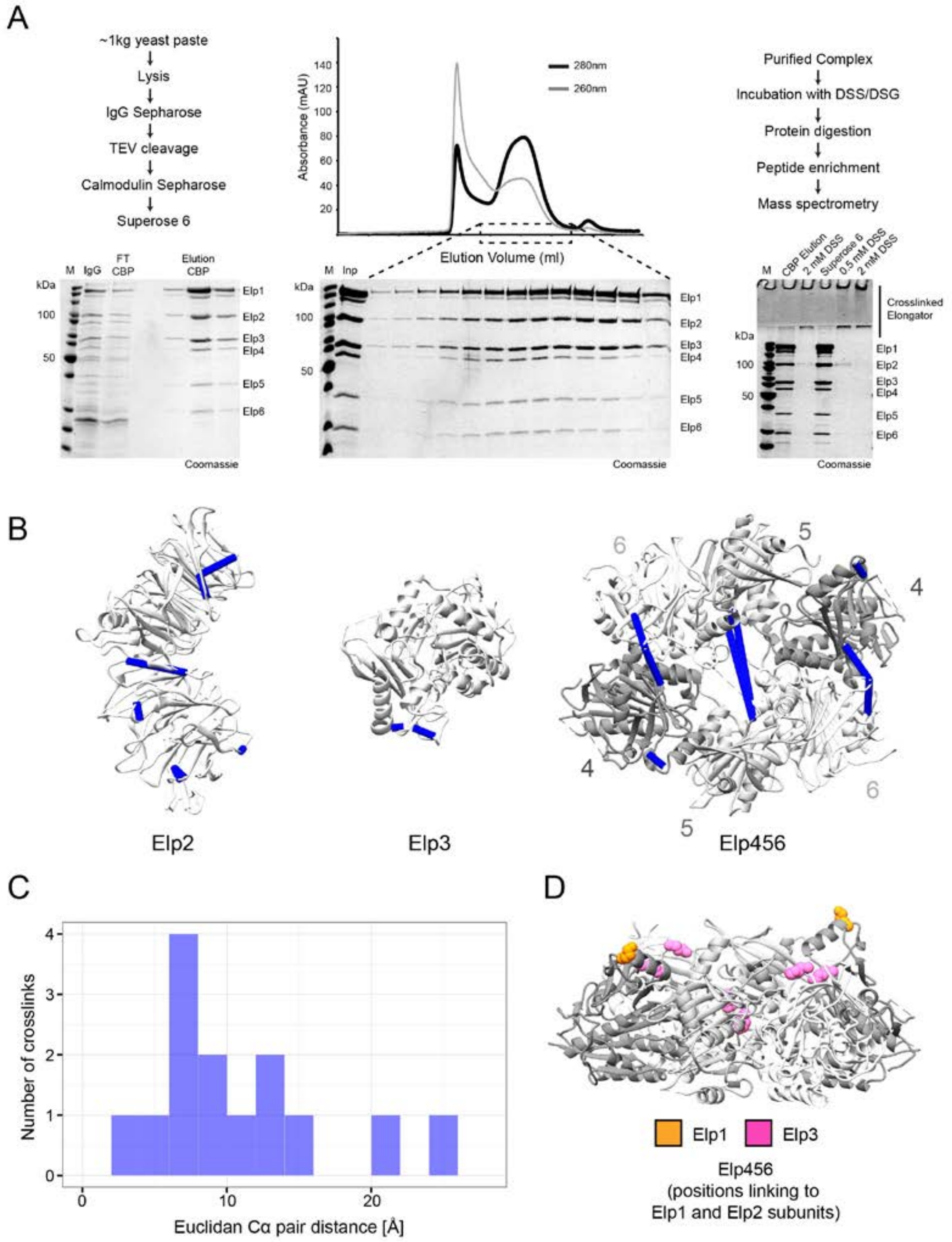

Appendix Figure S1 │ Sample preparation and crosslinking validation. A. Schematic overview of the Elp1-TAP purification protocol and SDS-PAGE analyses of the initial purification steps (left panel). Samples were analyzed by SDS-PAGE and stained by Coomassie. Identities of respective proteins are indicated on the right. Gel filtration of purified Elp1-TAP tagged Elongator complex shows UV absorption profiles at 260nm and 280nm (middle panel). The respective fractions were analyzed by SDS-PAGE and stained by Coomassie. Identities of respective proteins are indicated. Schematic overview of the used XL-MS protocol and SDS-PAGE analyses of the samples before and after incubation with indicated amounts of crosslinking reagent (right panel). Gel was stained by Coomassie and identities of respective proteins are indicated on the right. **B**. Crosslinks (blue lines) identified in this work mapped to the subunit structures. Only high confidence crosslinks (LD score greater than or equal to 30) are displayed. **C.** Histogram of crosslink distances. All crosslinks satisfy the Cα-Cα distance threshold of 30 Å. **D**. Residues of the Elp456 sub-complex subunits crosslinking to Elp1 and Elp3 subunits (no residue crosslinks to Elp2). All crosslinking positions exclusively locate to one side of the hexamer, suggesting its orientation relatively to the Elp123 sub-complex.

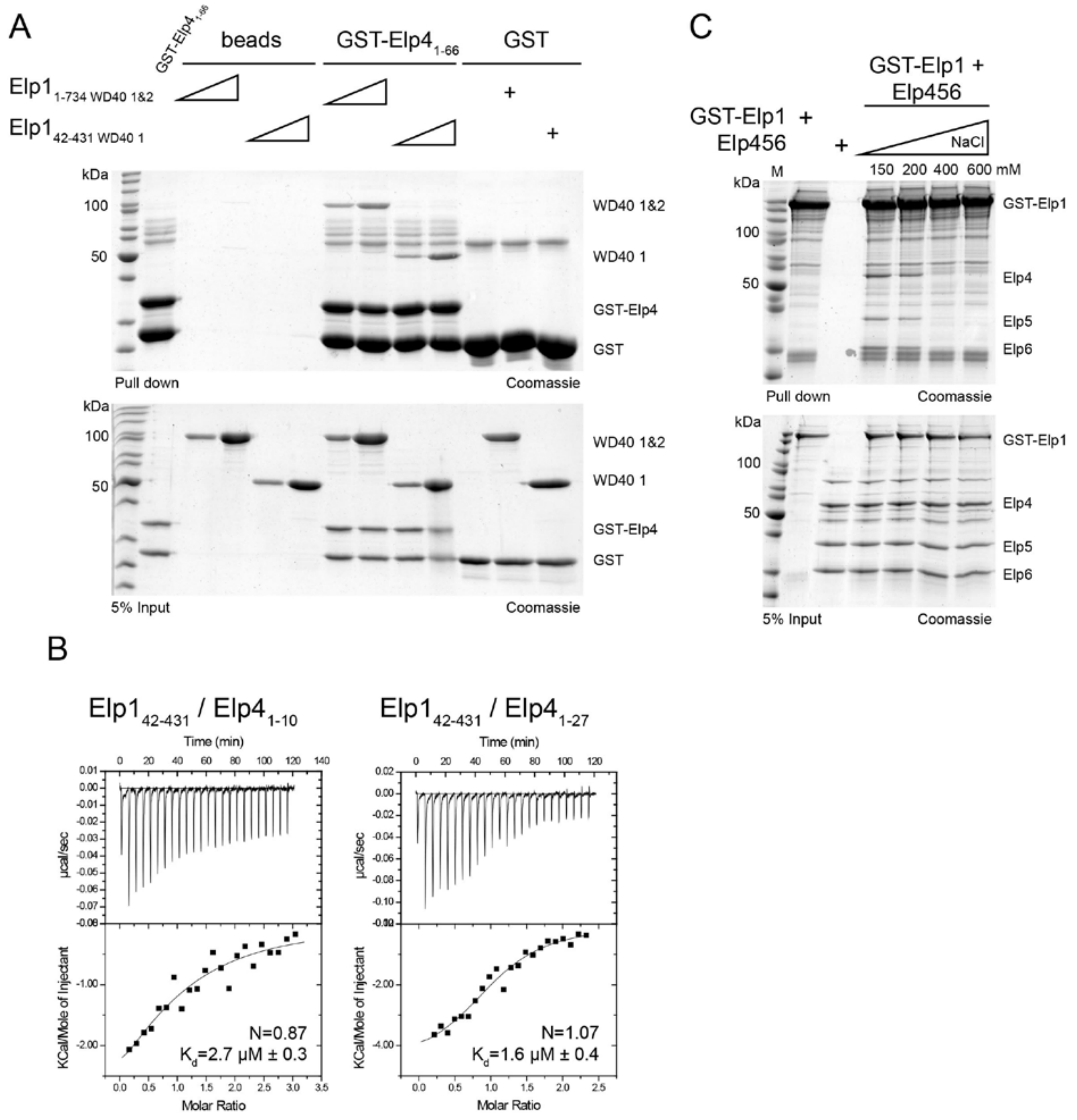

Appendix Figure S2 │ Validation of the Elp1-Elp3-Elp4 interaction network. A. GST-pull down assays of purified GST-tagged N-terminus of Elp4 with the two predicted untagged WD40 domains of Elp1 (1st WD40 aa 42-431 and both WD40s aa 1-734). GSH resin and untagged proteins were used as input controls. Lower panel shows 5% of the input and upper panel shows bound fractions. All samples were analyzed by SDS-PAGE and stained by Coomassie. Identities of respective proteins are indicated on the right. **B**. ITC measurement using the purified first WD40 domain of Elp1 (10 µM; cell) and synthetic peptides of the N-terminus of Elp4, aa1-10 and aa1-27 respectively (both 100 µM; syringe). Calculated Kd and stoichiometry (N) are indicated. **C**. Same as **A,** but using GST-tagged full length Elp1 in combination with untagged full length Elp456 in presence of increasing concentrations of NaCl.

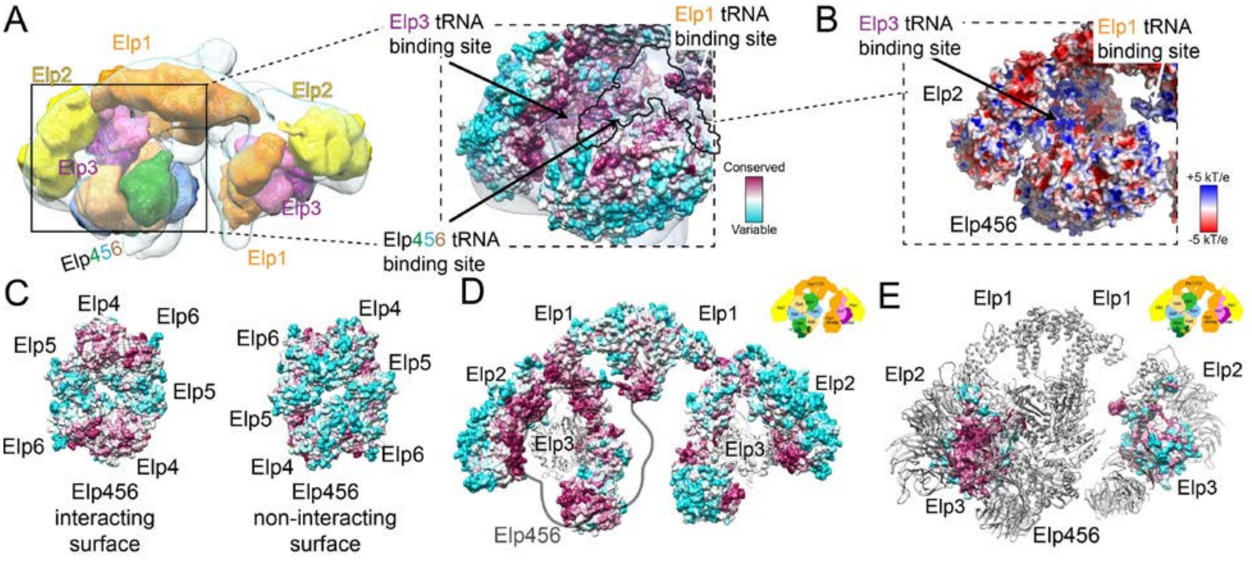

Appendix Figure S3 │ Analysis of surface properties of the holoElongator model. A. Sequence conservation of the tentative tRNA binding cleft mapped onto the molecular surface of the model. The full model is shown in a low-resolution representation within the negative stain envelope as viewed from the top (left). Sequence conservation is mapped onto the surface of the cleft as a color gradient (right). The black outline depicts a possible tRNA binding mode. **B.** Electrostatic potential mapped onto the surface of the cleft. Residues 74-94 and 374-405 of Elp3 (depicted as gray ribbons) were not used for calculating the charge distribution because their exact atomic structure is uncertain. Note also that the depicted electrostatic potential is only approximate due to low resolution of the model and missing regions, such as Arg-rich region of Elp1. **C.** Sequence conservation mapped onto the surface of Elp456 hexamer that interacts with Elp123 sub-complex (left) and the opposite, non-interacting surface (right). **D.** Sequence conservation mapped onto the surface of Elp1 and Elp2. Elp3 is shown in ribbon representation. For clarity, the Elp456 hexamer is depicted as an outline. The cartoon (top right) indicates the viewing angle. **E.** Sequence conservation mapped onto the surface of Elp3. All other subunits are shown in ribbon representation.

Appendix Table S1. Crystallographic data collection, phasing and refinement statistics

	Elp2 full length<br>SeMet<br>PDB ID 5M2N
**Data collection**	
Space group	$P6_122$
Cell dimensions	
a, *b*, *c* (Å)	79.69 79.69 532.15
α, β, γ (°)	90, 90, 120
Wavelength	0.97898
Resolution (Å) †	50-2.8 (2.89-2.8)
R_{meas}	14.3 (81.9)
$I/\sigma(I)$	14.40 (2.58)
$CC_{1/2}$	0.99 (0.68)
Completeness (%)	99.6 (95.7)
Redundancy	9.3 (7.2)
**Refinement**	
Resolution (Å)	50-2.8
No. reflections	45798
R_{work} / R_{free}	19.9/23.5
No. atoms	
Protein	5763
Ligand/ion	0
Water	23
B factors	
Protein	45.3
Ligand/ion	
Water	23.8
r.m.s deviations	
Bond lengths (Å)	0.002
Bond angles (°)	0.561

[a] Values in parentheses are for highest-resolution shell.
[†] Resolution limits according to I/σ of 2 is are 2.7 Å for Elp2

Appendix Table S2. List of highly confident crosslinks identified in this work (ld-score at least 30)
Please see source data 1 for a complete list and additional specifications of the detected cross links

Protein 1	Protein 2	Residue 1	Residue 2	ld-Score
Elp6	Elp4	230	218	47.01
Elp3	Elp1	521	759	42.93
Elp1	Elp4	1223	218	42.93
Elp1	Elp3	510	473	43.22
Elp1	Elp3	1217	401	38.38
Elp1	Elp3	1223	401	38
Elp1	Elp4	1270	451	38
Elp4	Elp1	177	1217	36.66
Elp4	Elp3	399	61	36.96
Elp6	Elp3	225	401	36.92
Elp4	Elp3	399	14	38.22
Elp1	Elp3	220	473	40.6
Elp4	Elp3	399	6	33.17
Elp1	Elp4	190	218	32.95
Elp5	Elp3	140	6	32.62
Elp1	Elp3	1270	401	32.41
Elp1	Elp3	1223	61	32.18
Elp5	Elp3	140	14	35.3
Elp1	Elp4	198	218	37.14
Elp1	Elp4	1223	213	34.29
Elp2	Elp1	460	199	33.01
Elp4	Elp3	399	9	32.69
Elp1	Elp4	199	218	34.34
Elp1	Elp4	1217	213	32.96
Elp3	Elp4	342	370	30.46
Elp1	Elp3	1270	6	34.14
Elp4	Elp1	213	199	37.5
Elp2	Elp1	249	967	43.34
Elp1	Elp4	1223	177	39.77
Elp4	Elp3	143	401	33.98
Elp1	Elp3	1270	14	38.89
Elp1	Elp3	1223	14	31.29
Elp1	Elp4	1223	143	30.06
Elp1	Elp3	1217	19	32.56
Elp4	Elp1	186	199	32.36
Elp4	Elp1	176	756	30.34
Elp1	Elp1	1223	1270	45.86
Elp2	Elp2	249	257	44.11
Elp4	Elp4	235	143	43.75

Protein 1	Protein 2	Residue 1	Residue 2	ld-Score
Elp1	Elp1	1223	818	44.79
Elp4	Elp4	143	177	42.47
Elp1	Elp1	1308	1223	48.58
Elp2	Elp2	260	250	41.37
Elp4	Elp4	143	218	41.35
Elp3	Elp3	528	473	40.16
Elp1	Elp1	190	199	41.03
Elp3	Elp3	14	61	39.4
Elp4	Elp4	143	55	40.81
Elp4	Elp4	207	213	43.56
Elp1	Elp1	1119	1223	40.63
Elp1	Elp1	1246	1223	38.74
Elp1	Elp1	1246	1217	40.07
Elp3	Elp3	86	79	37.45
Elp3	Elp3	401	61	36.91
Elp1	Elp1	220	199	37.73
Elp1	Elp1	818	1217	41.38
Elp4	Elp4	143	5	42.95
Elp3	Elp3	19	61	36.28
Elp1	Elp1	1270	1217	42.12
Elp3	Elp3	325	401	35.69
Elp4	Elp4	143	63	39.78
Elp1	Elp1	1308	1217	40.02
Elp1	Elp1	1308	846	37.25
Elp3	Elp3	6	61	34.93
Elp4	Elp4	143	213	34.95
Elp3	Elp3	453	401	34.37
Elp3	Elp3	423	401	34.28
Elp1	Elp1	1223	756	33.78
Elp2	Elp2	650	680	35.49
Elp1	Elp1	1119	1217	35.11
Elp4	Elp4	177	218	33.99
Elp1	Elp1	818	846	38.2
Elp1	Elp1	1217	1217	33.3
Elp4	Elp4	177	211	32.83
Elp4	Elp4	211	218	33
Elp4	Elp4	177	207	32.8
Elp1	Elp1	1062	1223	36.13
Elp4	Elp4	207	218	35.86
Elp1	Elp1	220	198	32.12
Elp3	Elp3	401	14	32.09
Elp4	Elp4	186	218	31.78

Protein 1	Protein 2	Residue 1	Residue 2	ld-Score
Elp4	Elp4	169	177	34.62
Elp1	Elp1	1217	756	35.06
Elp5	Elp5	140	140	30.76
Elp3	Elp3	401	19	32.4
Elp4	Elp4	186	213	35.42
Elp1	Elp1	1119	1232	33.34
Elp4	Elp4	169	218	31.77
Elp6	Elp6	230	269	36.69
Elp2	Elp2	249	260	33.98
Elp2	Elp2	25	28	35.31
Elp2	Elp2	699	680	38.1
Elp1	Elp1	1223	1223	35.4
Elp4	Elp4	235	218	32.21
Elp4	Elp4	55	5	33.05
Elp1	Elp1	1062	1217	32.55
Elp1	Elp1	1316	1223	31.6
Elp1	Elp1	1217	759	31.57
Elp1	Elp1	1223	1217	36.7
Elp4	Elp4	177	213	34.82
Elp4	Elp4	169	143	35.43
Elp2	Elp3	257	86	34.49
Elp4	Elp4	55	218	33.58
Elp1	Elp1	1217	1232	32.96
Elp1	Elp1	1052	1223	32.4
Elp1	Elp1	1052	1217	32
Elp3	Elp3	401	473	35.71
Elp1	Elp1	1270	1232	30.84
Elp1	Elp1	1223	1232	30.69
Elp1	Elp1	510	1223	35.14
Elp1	Elp1	1223	759	38.53
Elp1	Elp1	1308	1232	34.04
Elp1	Elp4	1246	370	33.92
Elp3	Elp1	325	1217	33.13
Elp2	Elp3	249	79	31.81
Elp6	Elp4	230	399	30.89

Ld-score – linear discriminant crosslink confidence score as calculated by xQuest
