# Supplementary material for: Architecture of the yeast Elongator complex
Source: EMBO Rep. 2016 Dec 14;18(2):264–79. doi: 10.15252/embr.201643353 (PMC5286394; doi:10.15252/embr.201643353)
Supplement: Supplementary file 2 — Expanded View Figures PDF [file EMBR-18-264-s002.pdf]

Expanded View Figures

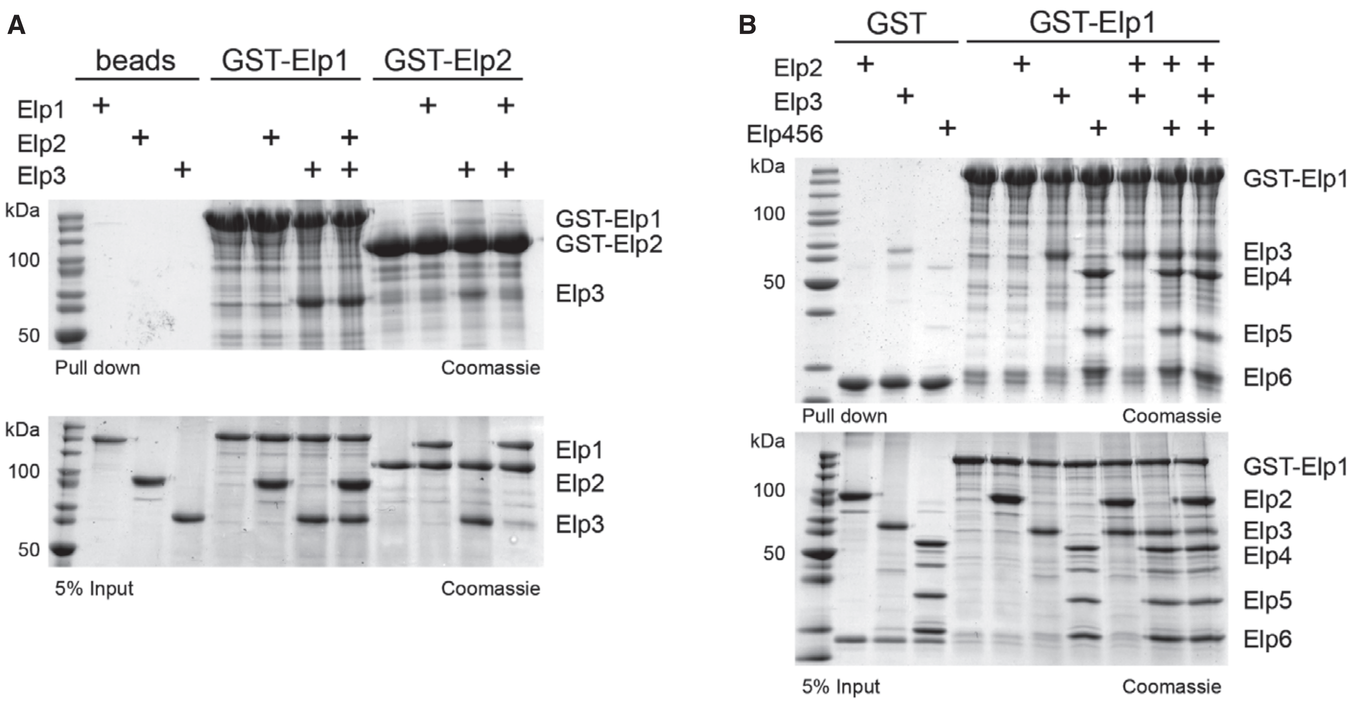

Figure EV1. Reconstitution of Elongator sub-complexes *in vitro* using individually purified subunits.

A GST pull-down assays of purified full-length GST-tagged Elp1 and Elp2 with untagged Elp2 and refolded and anaerobically reconstituted full-length Elp3. GSH resin and proteins were used as input controls. Lower panel shows 5% of the input and upper panel shows bound fractions. All samples were analyzed by SDS–PAGE and stained by Coomassie. Identities of respective proteins are indicated on the right.

B Same as (A), but using GST-tagged full-length Elp1 with combinations of Elp2, Elp3, and Elp456. GST with untagged proteins were used as input controls.

Figure EV2. EM analysis of holoElongator. ▶

A Fourier Shell Correlation curve for the holoElongator reconstruction. The red line indicates FSC = 0.143 and the green line shows FSC = 0.5.

B Euler plots for the angular distribution of holoElongator model in three different views.

C Fitting of Elp1-CTD dimer structure to arch of the holoElongator density in three different views. Panels show the best-scoring fits (top), a histogram of normalized EM cross-correlation scores (left), and a plot of *P*-values for all the solutions (right) and the top solutions (red arrow) in the inset. The *P*-values were calculated as described in Materials and Methods.

D Same as (C) using the mirrored holoElongator reconstruction.

E Tilt-pair validation of holoElongator. Tilt-pair validation plots are shown for holoElongator and RNA polymerase III particles imaged at 0° and 15° tilt angles. The two data sets of 117 and 197 particles were imaged on a FEI T12 microscope in sequence using identical microscope and detector settings. In addition, the obtained tilt pairs were also processed in parallel by the same validation workflow (Materials and Methods). The position of each point represents the difference of Euler angles due to tilting for a given particle pair plotted in polar coordinates by overlaying the scatter and the histogram plot. For both samples, most tilt pairs cluster at a tilt angle of approximately +15°.

© 2016 The Authors

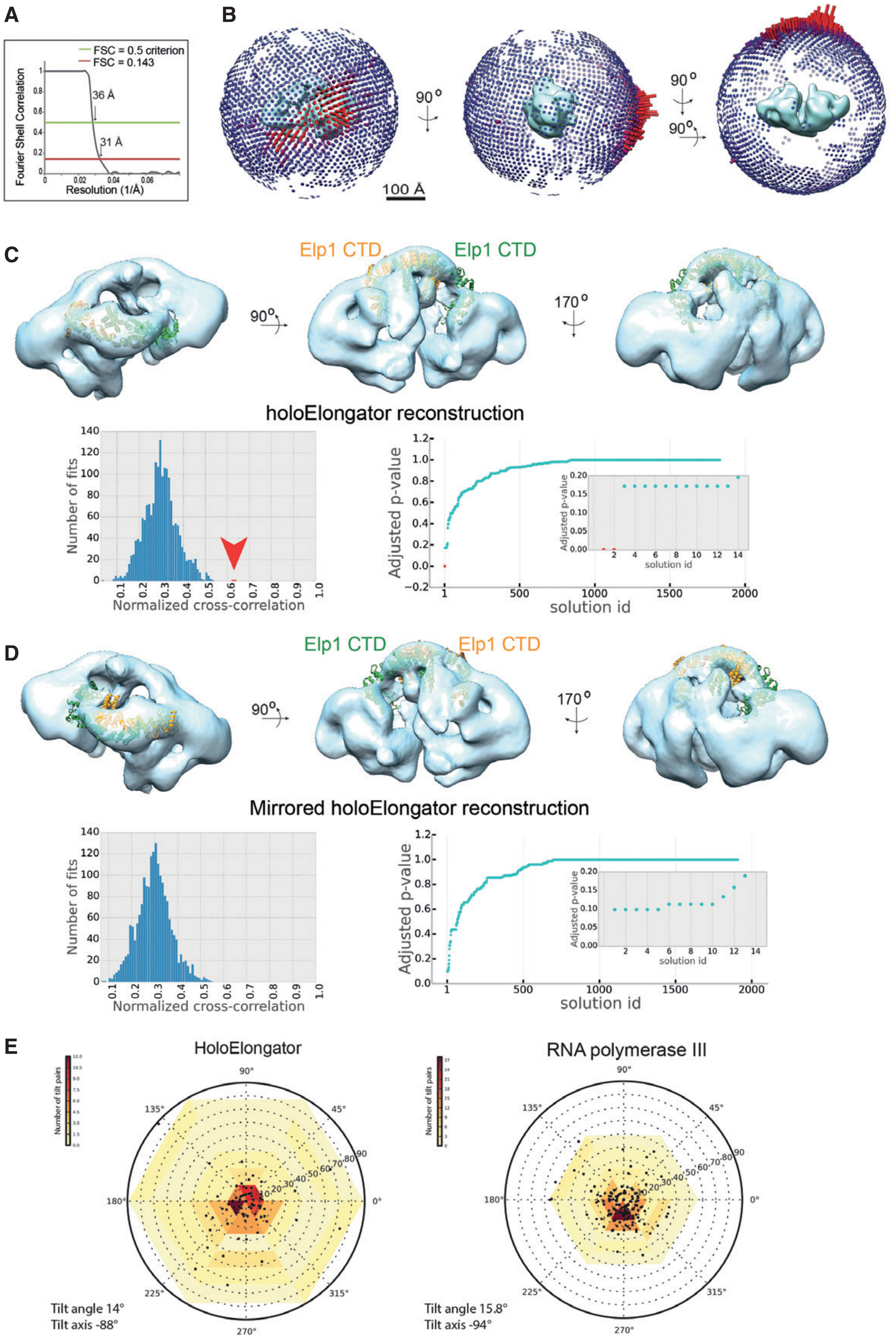
A
FSC = 0.5 criterion
FSC = 0.143
Fourier Shell Correlation
36 Å
31 Å
Resolution (1/Å)
B
90°
100 Å
C
Elp1 CTD
Elp1 CTD
170°
holoElongator reconstruction
Number of fits
Normalized cross-correlation
Adjusted p-value
solution id
D
Elp1 CTD
Elp1 CTD
Mirrored holoElongator reconstruction
E
HoloElongator
RNA polymerase III
Number of tilt pairs
Tilt angle 14°
Tilt axis -88°
Tilt angle 15.8°
Tilt axis -94°

Figure EV2.

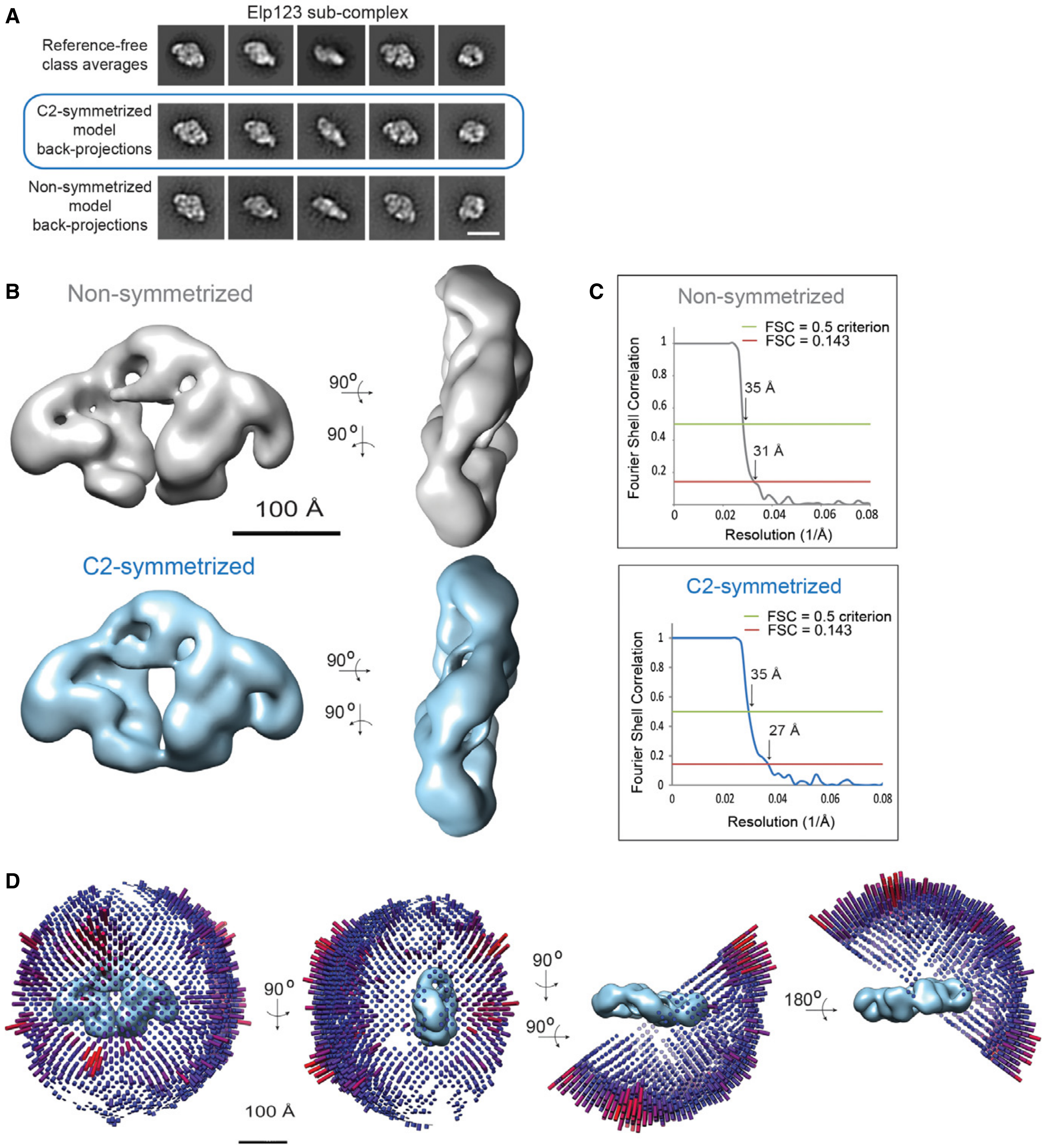

Figure EV3. EM analysis of the Elp123 sub-complex.

A Comparison between the Elp123 reference-free class averages (upper row), the back-projections of the C2-symmetrized Elp123 model (middle row) and the back-projections of the non-symmetrized Elp123 model (lower row). Scale bar, 20 nm.

B Comparison between the non-symmetrized (in gray) and the C2-symmetrized (in blue) Elp123 models in two different views. The scale bar corresponds to 100 Å.

C Fourier Shell Correlation curve for the non-symmetrized and the C2-symmetrized Elp123 models. The red line indicates FSC = 0.143 and the green line shows FSC = 0.5.

D Euler plots for the angular distribution of holoElongator model in four different views.

© 2016 The Authors

Figure EV4. Localization of Elp2 in the Elp123 sub-complex.

A Fitting of Elp1-CTD dimer structure to the arch of the Elp123 density and its mirror volume. Panels show the best-scoring fits (left), a histogram of normalized EM cross-correlation scores (middle), and a plot of *P*-values for all the solutions (right) and the top solutions in the inset. The *P*-values were calculated as described in Materials and Methods. The histogram and the *P*-value plot show that the best-scoring fit of Elp1-CTD to the Elp123 density is significantly better than other fits (marked in red and by red arrow).

B Gallery of low-pass-filtered images of the partial Elp123 sub-complex in top view (upper row) and comparison between the reference-free class averages (middle row) and the back-projections of the partial Elp123 model (lower row). Scale bar, 20 nm.

C EM reconstruction of the partial Elp123 sub-complex at 31 Å resolution. Scale bar, 100 Å.

D Fourier Shell Correlation curve for the partial Elp123 model. The red line indicates FSC = 0.143 and the green line shows FSC = 0.5.

E Superimposition of the full Elp123 model (depicted in mesh) and the partial Elp123 model (in green). The volumes were filtered to 35 Å for comparison. The difference density calculated by subtracting the partial from the full Elp123 complex is shown in purple.

F Structure of Elp2 at 2.8 Å in comparison with the published structure of Elp2 (PDB ID 4XFV) at 3.2 Å resolution. Structures are shown in cartoon representation and strands, helices, and subdomains are color-coded and labeled. A heat map of structural variance is shown on the right.

© 2016 The Authors

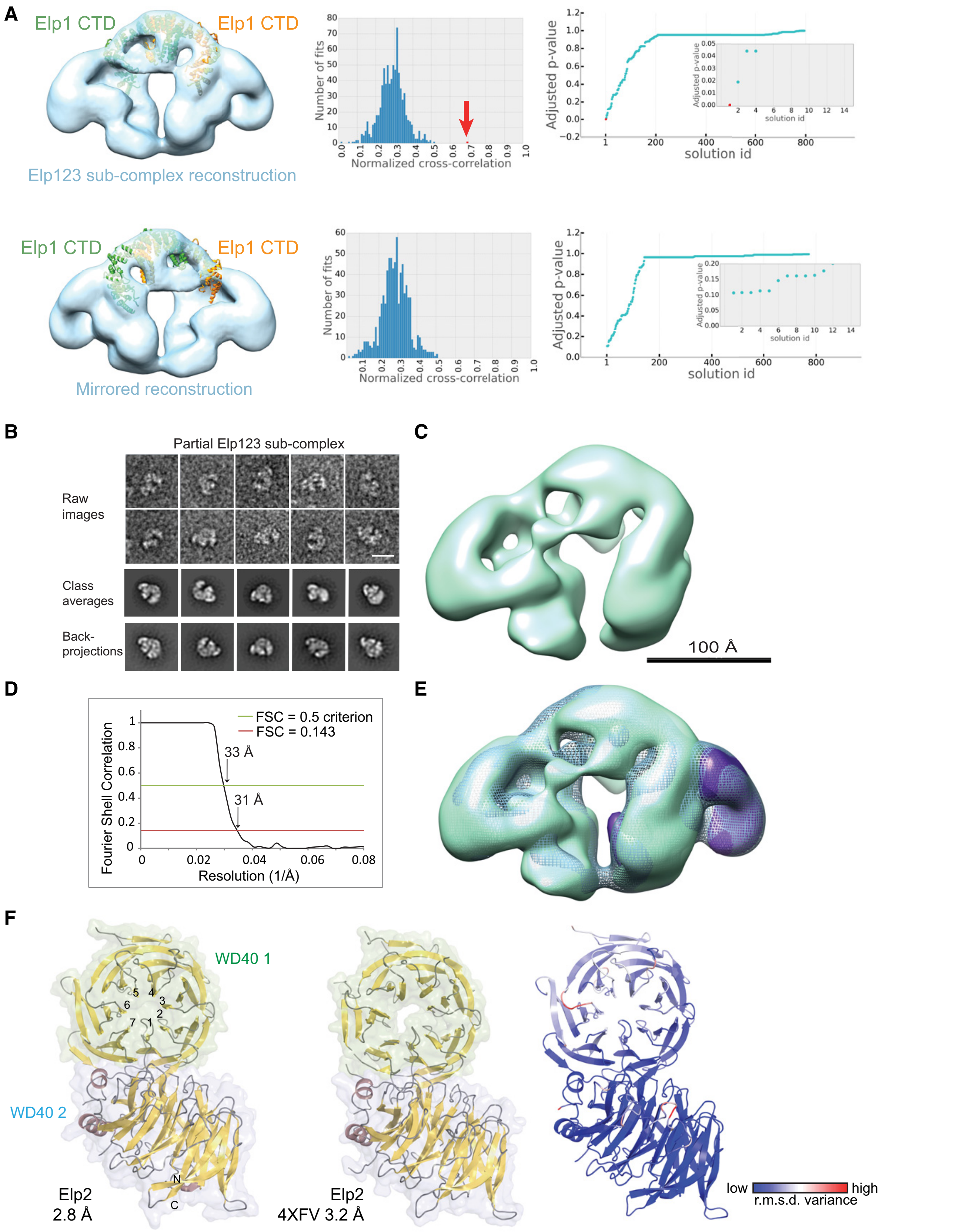
A
Elp1 CTD
Elp1 CTD
Elp123 sub-complex reconstruction
Number of fits
Normalized cross-correlation
Adjusted p-value
solution id
Elp1 CTD
Elp1 CTD
Mirrored reconstruction
B
Partial Elp123 sub-complex
Raw images
Class averages
Back-projections
C
100 Å
D
FSC = 0.5 criterion
FSC = 0.143
33 Å
31 Å
Fourier Shell Correlation
Resolution (1/Å)
E
F
WD40 1
WD40 2
Elp2
2.8 Å
Elp2
4XFV 3.2 Å
low
high
r.m.s.d. variance

Figure EV4.

A

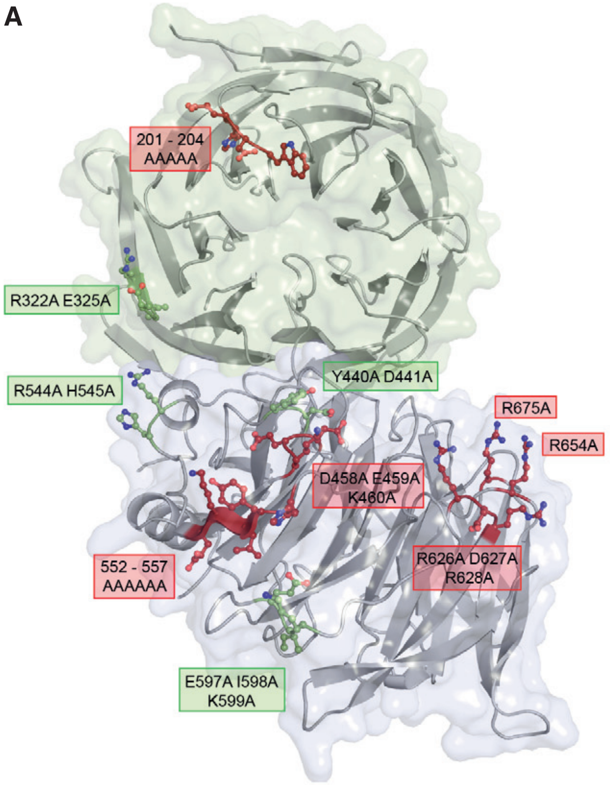

B

Position (aa)	Δelp2 SUP4 30°C	Δelp2 SUP4 30°C -ade	Δelp2 SUP4 37°C	Δelp2 SUP4 37°C -ade	Δelp2 Δurm1 30°C	Δelp2 Δurm1 37°C
Y440A D441A	white	+	white	+	+++	++
D458A E459A K460A	pink	-	pink	-	+++	++
552 - 557 AAAAAA	pink	-	pink	-	++	+
R626A D627A R628A	pink	-	pink	-	+	+/-
E597A I598A K599A	white	+	white	+	+++	++
201 - 204 AAAAA	pink	+/-	pink	-	+++	++
R544A H545A	white	+	white	+	+++	++
R654A	pink	-	pink	-	+++	++
R675A	pink	-	pink	-	++	+
R322A E325A	white	+	white	+	+++	+++
empty	pink	-	pink	-	++	+
WT	white	+	white	+	+++	+++
WT-tap	white	+	white	+	++	++

C

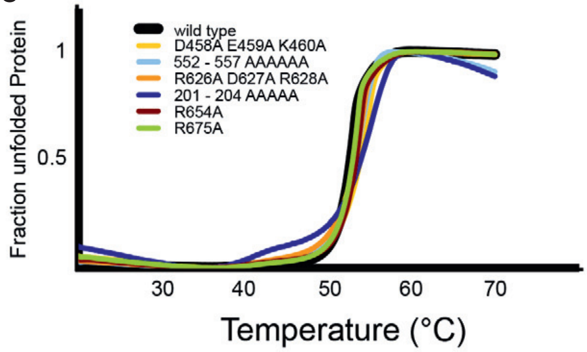

Figure EV5. Elp2 mutational analyses.

A Localization of tested surface mutants. Mutants showing a phenotypical response are labeled in red, and mutants showing weak or wild-type phenotypes are labeled in green.

B Summary of all tested mutants and associated phenotypes linked to altered tRNA modification.

C Comparative thermostability analyses of purified proteins of wild-type Elp2 and indicated mutants thereof.

 © 2016 The Authors
